# *Cryptosporidium* and other intestinal parasitic infections among HIV patients in southern Ethiopia: significance of improved HIV-related care

**DOI:** 10.1186/s13071-016-1554-x

**Published:** 2016-05-10

**Authors:** Techalew Shimelis, Yayehyirad Tassachew, Tariku Lambiyo

**Affiliations:** Department of Medical Laboratory Science, Hawassa University, P. O. Box 1560, Hawassa, Ethiopia

**Keywords:** *Cryptosporidium*, Intestinal parasites, HIV/AIDS, Ethiopia

## Abstract

**Background:**

Intestinal parasitic infections are known to cause gastroenteritis, leading to higher morbidity and mortality, particularly in people living with HIV/AIDS. This study aimed to determine the prevalence of *Cryptosporidium* and other intestinal parasitic infections among HIV patients receiving care at a hospital in Ethiopia where previous available baseline data helps assess if improved HIV-related care has reduced infection rates.

**Methods:**

A cross-sectional study was conducted at Hawassa University Hospital in southern Ethiopia from May, 2013 to March, 2014. A consecutive sample of 491 HIV- infected patients with diarrhea or a CD4 T cell count < 200 cells/μl were prospectively studied. A single stool sample was collected from each study participant and processed using direct, formol-ether concentration, and modified Ziehl-Neelsen techniques for the diagnosis of *Cryptosporidium* and other intestinal parasites. The study was approved by the Institutional Review Board of the College of Medicine and Health Sciences, Hawassa University. Physicians managed participants found to be infected with any pathogenic intestinal parasite.

**Results:**

The overall prevalence of intestinal parasitic infections among the study population was 35.8 %. The most prevalent parasites were *Cryptosporidium* (13.2 %), followed by *Entamoeba histolytica/dispar* (10.2 %), and *Giardia lamblia* (7.9 %). The rate of single and multiple infections were 25.5 and 10.3 %, respectively. Patients with a CD4 T cell count < 200 cells/μl had a similar rate of any intestinal parasitic infection or cryptosporidiosis compared to those with counts ≥ 200 cells/μl, but with some type of diarrhea.

**Conclusion:**

The study shows high prevalence of intestinal parasitic infections in the study population. However, the results in the current report are significantly lower compared to previous findings in the same hospital. The observed lower infection rate is encouraging and supports the need to strengthen and sustain the existing intervention measures in order to further reduce intestinal parasitic infections in people living with HIV/AIDS.

## Background

*Cryptosporidium* and other parasitic infections are known to cause gastroenteritis and malnutrition, which ultimately leads to significant morbidity and mortality, particularly in people living with human immunodeficiency virus/acquired immunodeficiency syndrome (HIV/AIDS). Studies have shown that about 30–60 % of AIDS patients in developed countries and 90 % in developing countries experience diarrhea [1]. Bacteria, parasites, fungi and viruses are known etiologic agents of diarrhea, although those of parasitic origin are most prominent in patients with AIDS in developing countries [2].

A group of parasites that came into prominence in humans with the emergence of HIV infection are among known pathogens that cause diarrhea [3]. In association with progressive decline in immunological responses in HIV-infected patients, the epidemiology of these parasites has been intensified. Previous studies showed that HIV patients, particularly those with a lower CD4 T cell count, have higher prevalence of opportunistic intestinal parasites such as *Cryptosporidium* species, *Isospora belli*, *Cyclospora cayetanensis*, microsporidia, and *Strongyloides stercoralis* [4, 5].

*Cryptosporidium* is a ubiquitous coccidian parasite infecting humans and a wide range of domestic and wild animals [6]. The parasite is robust to various chemicals and environmental stresses, lacks host-specificity, has a direct life-cycle and can propagate by autoinfection, is excreted in large numbers with faeces, and has a low infectious dose; these factors attribute for its spread and ubiquity in the environment [6, 7]. Transmission of *Cryptosporidium* is mainly through the faecal-oral route, as well as through contaminated water and food, person-to-person spread and contact with infected animals [8]. This parasite infection in immunodeficient patients may result in severe and chronic diarrhea, dehydration, wasting and death [9, 10]. As no effective therapy is currently available, the need to prioritize interventions that decrease the risk of acquiring *Cryptosporidium* infection have to be emphasized. In this regard, early initiation of antiretroviral therapy (ART) in HIV infected patients helps restore immunity and prevents acquisition and/or facilitates clearance of an established infection [11].

In Ethiopia, most health facilities that provide HIV care and treatment services routinely screen all HIV-infected persons for non-opportunistic intestinal parasites at enrolment in the ART clinic as well as those with clinical indications during follow-up. However, opportunistic intestinal parasites are poorly addressed and no routine screening service is provided. We previously reported the high prevalence of protozoal and helminthic infections among HIV-infected people in southern Ethiopia where the infection rates for *Cryptosporidium* and *S. stercoralis* were shown to be up to 25 and 12.6 %, respectively [5, 12].

Improved care and treatment provided to people living with HIV/AIDS (PLHIV) has greatly decreased HIV-related morbidities and mortalities in developing countries. Currently, in Ethiopia, HIV-infected persons initiate ART at CD4 T cell count < 500 cells/μl compared to < 200 cells/μl and < 350 cells/μl in the year before 2009 and 2013, respectively. Moreover, intervention measures have been undertaken in order to reduce the risk of infection with intestinal parasites and decrease their clinical consequences in HIV-infected people. Since the year 2013, PLHIV have been provided with household water treatment (sodium hypochlorite solution with the brand name Wuha Agar and a flocculent-disinfectant product with the brand name PuR), safe water storage using a narrow-mouth container with tap, soap, anti-helminthic drugs and oral rehydration salt free of charge. Whether such interventions have reduced the public health significance of opportunistic or other intestinal parasites has yet to be systematically evaluated. This study was, therefore, conducted to determine the prevalence of *Cryptosporidium* and other intestinal parasitic infections among HIV-infected patients who receive care at one of the largest hospitals in southern Ethiopia where previous available baseline data helps evaluate if improved HIV-related care has reduced the rate of infection.

## Methods

A cross-sectional study was conducted at Hawassa University Hospital from May, 2013 to March, 2014. The hospital is situated in Hawassa, the Capital City of the Southern Nations, Nationalities and Peoples’ Regional State in Ethiopia. It is the largest hospital in the administrative region and provides care and treatment to 6230 HIV-infected people. Clinical and laboratory investigations including immunological (CD4 T cell count) and/or biochemical assessments at enrolment and on three-monthly follow-up visits help determine patient’s eligibility for ART as well as monitor response to treatment.

The study population consisted of consecutive HIV-infected individuals who monitor their disease status at the ART clinic of the Hospital and with a CD4 T cell count < 200 cells/μl and/or with a complaint of diarrhea. A total of 491 individuals that fulfilled the specified inclusion criteria were prospectively recruited. Persons who took anti-parasitic treatment within a month prior to the time of data collection were excluded.

Counselor nurses interviewed the study participants collected socio-demography, risk factor, current symptom and duration of diarrhea data using structured questionnaires. Diarrhea was defined as watery or loose stool passed at least three times over a 24-h period. The persistence of diarrhea for more than 14 days was defined as chronic; while, diarrhea that lasts less than 14 days was acute. All participants provided a single stool sample on the same day of interview and measurement of CD4 T cells. The stool samples were processed using direct microscopy (normal saline and iodine preparations) and formol-ether concentration techniques to detect trophozoite/cyst, larvae and ova of intestinal parasites as appropriate. The modified Ziehl–Neelsen staining technique was performed to detect oocysts of intestinal coccidian parasites, as described elsewhere [13]. In brief, air-dried stool smears were fixed with methanol for 3 min, stained by carbol fuchsine for 15 min, decolorized with 1 % acid alcohol for 15 s, and counterstained with 0.5 % methylene blue for 30 s. Stained smears were air-dried and examined microscopically (using 100× objective) for oocysts.

The current data were compared with previous data collected from November, 2008 to March, 2009. During this period, HIV-infected patients had been initiating ART at a CD4 level < 200 cells/μl. In both studies, single stool samples were collected from the study participants and analyzed using the same laboratory techniques. HIV-infected individuals enrolled in the previous study were naïve for ART and regardless of diarrheal status or level of CD4 T cell count. In comparison, the present study investigated only patients with a CD4 T cell count < 200 cells/μl and/or with a complaint of diarrhea. It was shown in the previous study that these subgroups were more affected by opportunistic or any intestinal parasitic infections [5].

Data entry and analysis was performed using SPSS Version-16. Descriptive summaries were presented in terms of mean, range and proportions as appropriate. Differences between proportions were evaluated using Pearson’s Chi-square test (*χ*^2^), and a *P*-value < 0.05 was considered significant.

The study was approved by the Institutional Review Board of the College of Medicine and Health Sciences, Hawassa University. Participation was voluntary and informed written consent was obtained from each study participant. Participants were informed of the confidentiality of their personal data, and physicians managed those found to be infected with any pathogenic intestinal parasite.

## Results

A total of 491 HIV-infected individuals were enrolled during the study period. Most study participants were urban residents (92.9 %) and females (60.7 %). The mean age of study participants was 33.1 years (range, 15–70; SD, 9.51). Concerning ART status, 55.8 % of the participants were on ART and the remaining 44.2 % were ART-naïve (Table 2). Individuals with a level of CD4 T cell count < 200 cells/μl accounted for 56.2 % of the participants. The remaining participants (43.8 %) were those who reported some type of diarrhea (acute or chronic). Of the participants with a CD4 level < 200 cells/μl, the majority (65.2 %) reported no diarrhea, while 18.1 and 16.7 % experienced acute and chronic diarrhea, respectively (Table 3).

**Fig. 1:**
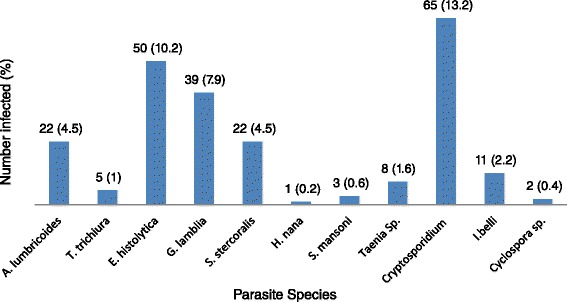
Prevalence of different species of intestinal parasites in the study population in Hawassa University Hospital, southern Ethiopia, 2013–2014

**Fig. 2:**
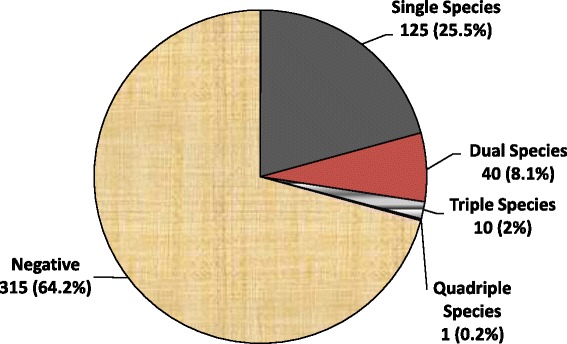
Prevalence of single and multiple parasite infections in the study population in Hawassa University Hospital, southern Ethiopia, 2013–2014

The cumulative prevalence of intestinal parasitic infections in the study population was 35.8 % (176/491). Individuals infected with protozoans accounted for 28.1 % of the study participants while 11 % were infected with helminths. The most prevalent parasites were *Cryptosporidium* (13.2 %), followed by *Entamoeba histolytica/dispar* (10.2 %) and *Giardia lamblia* (7.9 %). The same rate of infection (4.5 %) was observed for *Ascaris lumbricoides* and *Strongyloides stercoralis* while the rate for *Isospora belli* was 2.2 %. The species-wise prevalence of different intestinal parasites in the study population is presented in Fig. 1. The rates of single and multiple parasitic infections in the study population were 25.5 % and 10.4 %, respectively (Fig. 2).

The rate of intestinal parasitic infection was higher among rural residents (45.7 %), participants in the age range 40–49 years (44.3 %), males (36.3 %), and in those who had no work (41.8 %). Nonetheless, only educational level of the participants was found to be significantly associated with rate of intestinal parasitic infection (*χ*^*2*^ = 18.03, x*df* = 3, *P* < 0.001) where illiterates were found to be more affected (50.6 %) (Table 1).

**Table 1:** Intestinal parasitic infection in relation to socio-demography in the study population in Hawassa University Hospital, southern Ethiopia, 2013–2014

Characteristics	Number (%) tested	Number (%) positive for any parasite	*χ* ^*2*^	*P*-value
Residence				
Rural	35 (7.1)	16 (45.7)	1.53	0.22
Urban	456 (92.9)	161 (35.3)		
Age group (years)				
15 – 19	19 (3.9)	7 (36.8)	5.32	0.26
20 – 29	165 (33.6)	63 (38.2)		
30 – 39	186 (37.9)	58 (31.2)		
40 – 49	88 (17.9)	39 (44.3)		
≥ 50	33 (6.7)	10 (30.3)		
Sex				
Male	193 (39.3)	70 (36.3)	0.07	0.94
Female	298 (60.7)	107 (35.9)		
Occupation				
Government employee	73 (14.9)	23 (31.5)	6.55	0.623
Non-governmental employee	47 (9.6)	11 (23.4)		
House wife	133 (27.1)	55 (41.4)		
Student	13 (2.6)	5 (38.5)		
Merchant	85 (17.3)	31 (36.5)		
Housemaid	28 (5.7)	10 (35.7)		
No work	55 (11.2)	23 (41.8)		
Other	57 (11.6)	19 (33.3)		
Educational status				
Illiterate	89 (18.1)	45 (50.6)	18.01	< 0.0001
Primary	143 (29.1)	60 (42)		
Secondary	198 (40.3)	56 (28.3)		
Tertiary	61 (12.4)	16 (26.2)		

The majority of the study participants used pipe water for both domestic and laundry purposes (95.5 %) and reported no contact with animals (74.3 %). The rate of *Cryptosporidium* infection among participants in the age group 15–19 years, ART-naïve, had animal contact, and used pipe water were 15.8, 15.2, 15.1 and 13.6 %, respectively. However, none of these factors was found to be associated with *Cryptosporidium* infection (Table 2).

**Table 2:** Distribution of *Cryptosporidium* infection in relation to risk factors in the study population in Hawassa University Hospital, southern Ethiopia, 2013–2014

Characteristics	Number (%) tested	Number (%) positive for *Cryptosporidium*	*χ* ^*2*^	*P*-value
Water source				
Pipe	471 (95.9)	64 (13.6)	1.31	0.52
Well	17 (3.5)	1 (5.9)		
Lake	3 (0.6)	0 (0.0)		
Animal contact				
Yes	126 (25.7)	19 (15.1)	0.5	0.48
No	365 (74.3)	46 (12.6)		
ART status				
ART naïve	217 (44.2)	33 (15.2)	1.31	0.25
On ART	274 (55.8)	32 (11.7)		
Age group (years)				
15 – 19	19 (3.9)	3 (15.8)	0.84	0.93
20 – 29	165 (33.6)	19 (11.5)		
30 – 39	186 (37.8)	26 (14.0)		
40 – 49	88 (17.9)	13 (14.8)		
≥ 50	33 (6.7)	4 (12.1)		

The distribution of any intestinal parasitic infection or cryptosporidiosis in relation to diarrheal status and CD4 T cell count is presented in Table 3. The majority (65.2 %) of the study participants with a CD4 T cell count lower than 200 cells/μl reported no diarrhea. Participants with the lower CD4 T cell count had a similar rate of any intestinal parasitic infection (36.2 %) and cryptosporidiosis (15.6 %) compared to those with a CD4 count ≥ 200 cells/μl, but with some type of diarrhea (parasitic infection, 35.6 %; cryptosporidiosis, 10.2 %). In participants categorized by a CD4 T cell count, the rate of parasitic infection was higher in patients with chronic diarrhea. However, a statistically significant difference in rate of any parasitic infection by type of diarrhea was observed in a CD4 category 200–499 cells/μl (acute diarrhea 26.2 % *vs* chronic diarrhea 45.2 %; *χ*^*2*^ = 4.99, *df* = 1, *P* = 0.03). Similarly, difference in rate of *Cryptosporidium* infection by diarrhea status was statistically significant in patients with a CD4 level < 200 cells/μl (no diarrhea 15 %, acute diarrhea 6 %, chronic diarrhea 28.3 %; *χ*^*2*^ = 9.12, *df* = 2, *P* = 0.01).

**Table 3:** Distribution of any parasite and *Cryptosporidium* infections in relation to diarrheal status and CD4 T-cell count in the study population in Hawassa University Hospital, southern Ethiopia, 2013–2014

CD4+ count/μl	Diarrhea status	No. tested (%)	No. positive for any parasite (%)	*P*-value	No. positive for *Cryptosporidium*	*P*-value
< 200	No	180(65.2)	63(35)	0.16	27(15)	0.01
	Acute	50(18.1)	15(30)		3(6)	
	Chronic	46(16.7)	22(47.8)		13(28.3)	
	Total	276(56.2)	100(36.2)		43(15.6)	
200 – 499	Acute	103(71)	27(26.2)	0.03	7(6.8)	–
	Chronic	42(29)	19(45.2)		9(1.4)	
≥ 500	Acute	56(80)	24(42.9)	0.63	5(8.9)	–
	Chronic	14(20)	7(50)		1(7.1)	
	Total	215 (43.8)	77(35.8)		22(10.2)	

## Discussion

In this cross-sectional study, the cumulative prevalence of intestinal parasitic infection in the study population was found to be 35.8 %, which is comparable with a result reported in eastern Ethiopia (33.7 %) [14]. However, the current finding is significantly lower compared to a previous study in the same hospital (59.8 %) [5] or another study in Bahirdar (northwest Ethiopia) (69 %) [15]. The lower rate of infection in the current report is likely to be the outcome of improved care and treatment provided to people living with HIV/AIDS. In light of the relatively high risk population investigated in this study, the result is encouraging and emphasizes the importance of sustaining such intervention efforts.

As to the diversity of parasite species, several reports in Ethiopia showed similar results where helminths like *A. lumbricoides* and *S. stercoralis* and protozoan parasites such as *Cryptosporidium*, *E. histolytica*/*dispar* and *G. lamblia* were dominating species in the study population [5, 12, 14, 15]. However, compared to our previous study in the same hospital [5], the current prevalence of the specified parasites has decreased considerably: *A. lumbricoides* (12.2 *vs* 4.5 %) and *S. stercoralis* (12.6 *vs* 4.5 %). The rate of *S. stercoralis* infection in the current study was also lower than previous findings in Ethiopia: 12 % in Yirgalem (southern Ethiopia) [12], 10.7 % in Gondar (northern Ethiopia) [16], and 7.8 % in Jimma (southwestern Ethiopia) [17]. Similarly, protozoan parasites were detected at lower rates in the current study compared to the previous study conducted in the same hospital: *E. histolytica/dispar* (previous 24.8 *vs* current 10.2 %) and *G. lamblia* (11.2 *vs* 7.9 %). The decreased rate of cryptosporidiosis (50.1 *vs* 15.6 %) and isosporiasis (12.2 *vs* 2.2 %) among patients with a CD4 T cell count < 200 cells/μl may indicate the success of intervention efforts in reducing the significance of opportunistic intestinal parasitic infections in the specified high risk group*.* However, in contrast to our current report, the rate of cryptosporidiosis (26.9 %) among HIV-infected patients in Addis Ababa (central Ethiopia) was higher, which may partly be due to the sensitive laboratory technique (PCR-RFLP analysis) employed in the study [18]. Overall, the decreased rate of infection for different parasites may reflect the impact of the current intervention measures on various type of parasites with clinical and public health significance in HIV-infected people. It was possibly due to the decreased rate of occurrence of various parasitic species that the rate of multiple infections (10.4 %) in our current study was lower compared to that reported in our previous one (27.2 %) [5] or another study elsewhere (93.1 %) [15]. This report, therefore, encourages public health officials and other concerned bodies to further strengthen the existing HIV-related care and treatment program so that parasite-associated morbidity and mortality can be diminished.

Gastroenteritis is one of the major HIV-related illnesses and intestinal parasites are the most important agents implicated. We previously reported the significance of either HIV or parasitic infections independently or as co-infections to cause any type of diarrhea or chronic diarrhea [5]. It is routine clinical practice to screen patients with diarrhea for intestinal parasitic infections, although opportunistic parasites are usually overlooked in our context where laboratory services are limited. A similar rate of either any parasitic infection or cryptosporidiosis in patients with a CD4 T cell count < 200 cells/μl compared to those with a CD4 count ≥ 200 cells/μl, but with some type of diarrhea highlights the importance of providing a screening service for all patients with a CD4 count < 200 cells/μl regardless of their diarrheal status. A higher rate of parasitic infection was observed among patients that had chronic diarrhea, although the difference was found to be statistically significant only in the CD4 T cell count category 200–499 cells/μl. Similarly, the association of *Cryptosporidium* infection with diarrheal status was found to be statistically significant in patients with a CD4 level < 200 cells/μl in which the parasite was more likely to be detected in those who had chronic diarrhea. These results indicate that the chance of detecting intestinal parasites is higher among patients with chronic diarrhea. The detection rate of *Cryptosporidium* parasite was also significantly higher among patients with lower CD4 level, but with chronic diarrhea.

Various factors such as animal-human mixing patterns, access to safe drinking water, immunity and ART status of the study population have been reported to influence the epidemiology of cryptosporidiosis [7, 8, 11]. In agreement, reports in Ethiopia showed a lower rate of cryptosporidiosis among HIV-infected patients on ART compared to those who were naïve for ART [14, 19]. As the aim of the current investigation was to assess the status of intestinal parasitic infections in the context of improved HIV-related care, no attempt was made to establish matching subgroups for making valid comparisons. It is, therefore, difficult to reason why the rate of cryptosporidiosis was not found to be associated with factors including ART status, contact with animals and source of water in the current investigation. Which specific interventions or factors impacted the prevalence of parasitic infections needs to be investigated.

This study had some limitations in light of which its results need to be interpreted. First, as a single stool specimen was examined, the study did not benefit from examining multiple samples, which could improve the rate of detection. Secondly, the modified Ziehl-Neelsen staining technique has sensitivity inferior to methods such as polymerase chain reaction and direct fluorescent-antibody tests; thus, prevalence of cryptosporidiosis may be underestimated. Thirdly, incapability to further characterize isolates of *Cryptosporidium* deprives the possibility of defining the source of infection as well as routes of transmission in the study population. Lastly, the role of other diarrhea causing bacterial and viral pathogens was not addressed.

## Conclusion

The study shows a high prevalence of intestinal parasitic infections in the study population. However, the results are significantly lower compared to previous findings in the same hospital. The observed decreased rate of infection is most likely to be the outcome of intervention measures undertaken as part of improved HIV-related care in our health institution. Thus, it encourages the need to strengthen and sustain the existing intervention measures so as to further reduce the significance of intestinal parasitic infections in people living with HIV/AIDS. The current study also emphasizes the importance of screening HIV-infected patients for *Cryptosporidium* infection; with the priority given to patients with diarrhea as well as to those with a CD4 count < 200 cells/μl regardless of their diarrheal status.
